# Young Europeans – The Interplay between Short- and Medium-Term Development of European Identification Across Adolescence

**DOI:** 10.1007/s10964-025-02256-y

**Published:** 2025-09-22

**Authors:** Anna-Maria Mayer, Beatrice Bobba, Philipp Jugert, Elisabetta Crocetti

**Affiliations:** 1 Catholic University Eichstätt-Ingolstadt, Eichstätt, Germany; 2 Utrecht University, Utrecht, the Netherlands; 3 University of Duisburg-Essen, Essen, Germany; 4 Alma Mater Studiorum University of Bologna, Bologna, Italy

**Keywords:** European identity, Latent class growth analysis, Short- and medium-term development, Identity processes, Adolescence

## Abstract

Identification with the European group bears important implications for adjustment, intergroup processes, and civic participation. However, little is known about its development across different time scales in adolescence. The current study examined short- and medium-term developmental trajectories of European identification across two age cohorts separately and conjointly. Participants were 1552 Italian adolescents (*M*_age_ = 15.69, *SD*_age_ = 1.22; 47.04% females) attending the 1st (52.30%) and 3rd (47.70%) year of high school. Results highlight general stability in the short- and medium-term, although younger and older adolescents differed in their developmental patterns. Youth’s age and short-term fluctuations in European identification were linked to unique medium-term trajectories. This research highlights the importance of combining social and developmental approaches to comprehensively study how social identification unfolds in adolescence.

## Introduction

Throughout their lives, people must address questions regarding their identity, such as “Who am I?” or “Which is my place within society?”. During adolescence, these questions become especially prominent (Erikson, 1950) when cognitive and biological maturation processes, as well as societal expectations, prompt youth to think about the person they want to become. Identity formation includes establishing strong and stable commitments across different personal domains (e.g., educational, relational) and defining oneself as a member of meaningful social groups (e.g., football club, nationality). While personal identity has been extensively tackled by longitudinal research (see for review Branje et al., 2021) and accounting for different time scales (e.g., Becht et al., 2021), less is known about the development of social identities (Crocetti et al., 2018). However, social identities are important not only for individuals’ adjustment (e.g., Albarello et al., 2021) but also for intergroup processes (e.g., Bobba et al., 2024a) and civic participation (e.g., Landberg et al., 2018). This is especially the case for identification with the supranational European group. Against the backdrop of rising support for far-right and nativist political movements, European identification can potentially function as an inclusive common ingroup identity (Gaertner et al., 1993) for youth living in Europe, which can help reduce ingroup favoritism and outgroup bias (e.g., Konings et al., 2023). Understanding how young people develop and consolidate their identification with the European group requires attention to processes unfolding across different timescales. Short-term processes may drive long-term change (Moeller et al., 2022), while strong, consolidated identifications may lead to greater stability over time, with fewer day-to-day fluctuations. Capturing this dynamic developmental process thus requires a multiscale approach (Kunnen & Sugimura, 2021). This study examined the development of identification with the European group in two age groups of Italian adolescents, considering development on a short- and medium-term timescale separately and conjointly.

### Social Identities and Their Development

Identification with the European group can be understood by adopting social identity (Tajfel & Turner, 1979; *social identity theory*) and self-categorization (Turner et al., 1987; *self-categorization theory*) approaches. In line with these perspectives, individuals categorize themselves based on one (or more) social group membership(s) (social categorization, e.g., perceiving oneself as European), and identify with those groups to varying degrees (social identification, e.g., identifying with the European group). Generally, a social identity is “the part of an individual’s self-concept which derives from his [or her] membership of a social group (or groups) with the value and emotional significance attached to that membership” (Tajfel & Turner, 1979, p. 63). Membership criteria of social groups (i.e., defining group characteristics) include subjective perceptions based on real or imagined common group attributes (Turner et al., 1987).

The formation of meaningful social identifications is a crucial task for adolescents. Through interpersonal and intergroup comparisons, adolescents can redefine who they are as members of relevant groups (e.g., friends or classmates; Bennett & Sani, 2004) within their social contexts (Crocetti et al., 2023). Despite its relevance, research on the development of social identification is still very limited. Within the social identity research stream, most studies used experimental and cross-sectional designs (Crocetti et al., 2023), and the limited longitudinal research that exists has mostly focused on the interaction of social and personal identity (e.g., Albarello et al., 2018; Sugimura et al., 2024) or on intergroup outcomes (e.g., Bobba et al., 2024a) over time.

Despite their limitations, previous longitudinal studies provide preliminary insights into the pathways of social identity development. Specifically, social identifications appear to move from concrete to abstract, following general developmental patterns in cognition. For instance, adolescents might first consolidate their identification with more concrete groups (e.g., with friends), which in turn paves the way to identification with more abstract groups (e.g., with humanity, Albarello et al., 2021). Additionally, studies on national and ethnic identifications suggest that social identifications tend to remain relatively stable throughout adolescence (e.g., Karataş et al., 2023).

All in all, patterns of developmental change and stability (i.e., mean-level changes and rank order stability, Mroczek, 2007) in social identifications remain mostly unexamined. Furthermore, limited attention has been paid to the interplay between short- and medium-term developmental processes. Fluctuations in the short-term, which might signify evaluation processes and reconsideration of social identifications (Bobba et al., 2024a), might be drivers of development in the longer term. Thus, tackling their interplay is important for understanding the development of identification with relevant social groups, among which the European one.

### Why Focus on Identification with the European Group?

There are several identifications with social groups one might consider (e.g., national or ethnic group), that play a crucial role in adolescents’ development. So why focus on European identification, which could be seen as relatively abstract and distant compared to more proximal group identifications (e.g., local or regional)? First, research suggests that although the European group might appear abstract and distant, early adolescents are already beginning to develop a sense of being European (Barrett, 2013). Moreover, a study conducted across eight EU countries demonstrated that a significant portion of adolescents and young adults (30% to >50%) identified to a similar extent with Europe and their nation or even identified more strongly with Europe (Landberg et al., 2018). This indicates that adolescents perceive the European group as a viable group identity.

Second, belonging to and identifying with relevant social groups, which includes the European one, is a core human need (Vignoles, 2011) and it is especially crucial in adolescence (Crocetti et al., 2023). Specifically, forming stable and strong social identifications can decrease identity uncertainty and contribute to adolescents’ psychosocial adjustment (Branje et al., 2021). Moreover, identification with proximal (e.g., classmates) and distal (e.g., humanity) groups was found to heighten the social well-being of youth (Albarello et al., 2021).

Third, previous research (e.g., Konings et al., 2023) highlights the capacity of European identification to function as a common ingroup identity due to its relatively more inclusive characteristics – compared to typically more ethnically defined national identities. This suggests that adolescents who categorize themselves as Europeans, instead or in addition to their specific nationalities or ethnicities, might have a feeling of we-ness and belonging together, even if their subgroup identities differ. Thus, identification with the European group is a relevant social identity in adolescence, contributing to positive outcomes at individual, intergroup, and societal levels.

### What is Known About the Development of European Identification?

Currently, only two studies have examined European identity development in adolescence. Both followed the research tradition of personal identity (Erikson, 1950; *psychosocial theory of development*). The first study examined European identity development by identifying four identity statuses (i.e., achievement, moratorium, diffusion, foreclosure; Marcia, 1966) and investigated their stabilities across one year among German and Czech youth (Jugert et al., 2021). Moderate to high stability was found implying little developmental change across one year.

The second study examined the development of European identity processes (i.e., commitment, in-depth exploration, reconsideration of commitment; Crocetti et al., 2008) across ten days and a year, and the interrelation of both time scales in a sample of early German adolescents (Mayer et al., 2024). Across one year, significant mean-level changes were found, which were accompanied by moderate to high stability. Over the course of ten days, identity processes were found to be stable, meaning negligible change and high stability. Results also highlighted that adolescents with stable short-term commitments were likely to maintain strong commitments over time (i.e., show less fluctuations in commitment; Klimstra & Schwab, 2021).

When evaluating the evidence of these studies, it is notable that both cover only a one-year period of development and include either one (Mayer et al., 2024) or no clear age groups (Jugert et al., 2021). Previous longitudinal studies on other identity domains with assessments every year or every few years show that there is considerable stability in identity development across adolescence and young adulthood (see Branje et al., 2021 for review on personal identity research). However, across longer observation periods, evidence suggests slow but systematic identity maturation (for a review see Meeus, 2018). Arguably, extending the observation period beyond a year might capture identity maturation in its full variety (e.g., increasing levels of identification).

Further, social identity processes for European identification across timescales remained unexamined. However, time and the interrelation of identity processes across different time scales should play a crucial role in the development of European identification (Lichtwarck-Aschoff et al., 2008; for empirical evidence, see Becht et al., 2021). On the short-term timescale, identity processes tend to be more in flux and are characterized by variability (de Ruiter & Gmelin, 2021). Conversely, on the longer-term timescale, identity processes are characterized by change and consolidation of identity tendencies and patterns. While longer-term processes emerge from short-term processes, the latter are constrained by the former (Lichtwarck-Aschoff et al., 2008). On both timescales, identity can be conceptualized as expressions (i.e., concrete experiences, feelings, actions, and interactions in context) or cognitive self-reflections (e.g., “I feel identified with my group”; de Ruiter & Gmelin, 2021).

Additionally, including more age groups offers the possibility to compare youth at different life stages and their reactions to developmental challenges and societal changes. Adolescence is characterized by profound biological, cognitive, and social changes (Wigfield et al., 2006). From early to late adolescence, youth increase in their ability to think abstractly, experience an increase in executive functions, and increasingly reflect on themselves and on complicated problems. These biological-cognitive changes are accompanied by the capacity to conceive of abstract groups. As adolescents increasingly engage in a wider variety of social experiences (i.e., engagement with more diverse groups of people), older adolescents become less naïve and positive in their judgement of people (Flanagan & Stout, 2010), which is associated with decreasing levels of social trust across adolescence (Crocetti et al., 2021). Another major task during adolescence encompasses identity formation and taking over expected societal roles. As such, identity uncertainty is expected to decrease in early to middle adolescence, while identity consolidation is expected to increase from middle to late adolescence (Meeus, 2016, 2018). In essence, analyzing multiple age groups could shed light on the influence of broader social and cultural shifts on developmental processes of identification at different stages of adolescence.

## Current Study

Despite its relevance for adolescents’ definition of the self and its implications for intergroup attitudes and civic participation, little is known about the development of European identification in different age groups and across multiple timescales. The current study aims to advance knowledge on the development of identification with the European group in three directions. First, it examined developmental patterns (i.e., mean-level changes and rank-order stability) of identification with the European group in the medium- (i.e., monthly/yearly) and short-term (i.e., daily) among younger (13-year-olds) and older (16-year-olds) adolescents. Mean levels of identification with the European group were expected to remain mostly stable, or display a slight increase, across both timescales (Hypothesis 1), while rank-order stability was expected to increase over time (Hypothesis 2). Second, this study aimed to examine the developmental interplay between medium-term changes and daily fluctuations. Participants with higher initial identification with the European group were expected to fluctuate less in their daily European identification (Hypothesis 3). Conversely, participants who fluctuated more were expected to show a steeper decrease in European identification (Hypothesis 4). Third, the current research examined whether variability in medium-term mean-level changes could be traced back to the existence of different groups of participants and whether adolescents’ age group and daily fluctuations could be associated with unique developmental trajectories of identification with the European group. Three groups with distinct developmental trajectories based on initial levels of European identification were expected (i.e., low, medium, high; Hypothesis 5). Moreover, participants in the high European identification trajectory were assumed to fluctuate less than participants in the low identification trajectory (Hypothesis 6). The study’s hypotheses and statistical analyses were preregistered (10.17605/OSF.IO/23DKQ).

## Methods

### Participants and Procedure

The present research was approved by the Ethics Committee of the Alma Mater Studiorum University of Bologna (Italy) as part of the IDENTITIES “Managing identities in diverse societies: A developmental intergroup perspective with adolescents” project, from which participants for the current study are drawn. The IDENTITIES project is a cohort-sequential longitudinal research involving adolescents from high schools in the northern part of Italy (i.e., the Emilia-Romagna region), their parents, and teachers. Schools were selected through a stratified (by track and level of urbanization) randomized method and principals were approached to present the project. Upon their approval, the study was presented to students and their parents who also received written information. Active consent from parents was obtained prior to their children’s participation. Active consent was also obtained from adolescents of age (18 and older), while younger adolescents provided their assent to participate in the project. Participation in the study was voluntary, and students could withdraw their consent at any time.

**Fig. 1:**
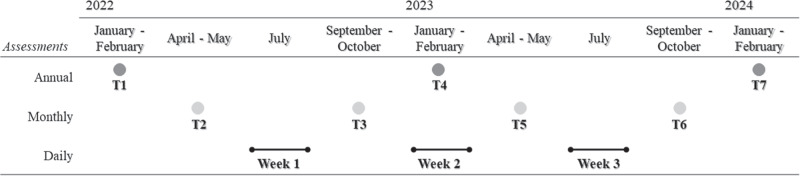
Timeline of study assessments. *Note*. T time

The current study focused on adolescents’ reports which were collected across several annual/monthly and daily assessments (see Fig. 1). Adolescents were required to create a personal code to ensure confidentiality and pair their answers over time. At each annual/monthly wave, researchers visited the participants in schools during class hours and assisted them during the online questionnaire completion (approx. 50–60 min). During the three weeks of daily assessments, adolescents received a brief online questionnaire (approx. 10 min) via e-mail. The first e-mail was sent during the late afternoon (i.e., 5:00 pm) and automatic reminders were scheduled at 7:30 and 9:00 pm for those who had not yet completed the daily assessment. The project started in January/February 2022 (T1) and followed youth from two age groups longitudinally, covering the entire span of adolescence, which in Italy coincides with the five years of high school.

A total of 1552 adolescents (*M*_age_ = 15.69, *SD*_age_ = 1.22; 47.04% females) were included. The first age group (52.30% of the sample) comprised adolescents who attended the 1st year of high school at T1 (i.e., around 14 years) and were followed until their 3rd year (i.e., approximately 16 at T7). The second group (47.70%) included adolescents who attended their 3rd year of high school at the beginning of the study (i.e., around 16 years of age) and were followed until their 5th year (i.e., around 18 years at T7). The sample included both ethnic majority (i.e., whose parents were both born in Italy; 79.87%) and ethnic minority (i.e., with at least one parent born outside Italy; 20.13%) youth. The latter mainly came from Eastern European countries. Further details on participants’ ethnic background and group comparisons are provided in document S1 of the Supplemental Materials.

Out of the total sample, 1547 participants (*M*_age_ = 15.69, *SD*_age_ = 1.22; 47.04% females; 52.30% 1st year students; 79.93% ethnic majority) completed at least one of the annual/monthly evaluations (T1-T7), while 774 adolescents (*M*_age_ = 15.63, *SD*_age_ = 1.15; 54.13% females; 51.62% 1st year students; 85.12% ethnic majority) completed at least one of the three weeks of daily assessments. Regarding parents’ education, most (47.56%) mothers had a medium (i.e., up to high school diploma), followed by those (32.08%) with a high (i.e., university degree or higher) and those (20.36%) with a low (i.e., up to middle school diploma) educational level. Among fathers, most (47.52%) had a medium, followed by those with a low (28.50%) and a high (23.98%) educational level. Information on sample attrition is reported in document S2 of the Supplemental Materials. The Little’s (1988) Missing Completely at Random (MCAR) test yielded a normed χ^2^ (χ^2^/df = 15,540.904/16,137) of 0.96, indicating that data were likely missing completely at random. Therefore, the total sample of 1552 participants was included in the analyses, and missing data were handled with the Full Information Maximum Likelihood (FIML) procedure available in M*plus* (Kelloway, 2015).

### Measures

#### Demographics

Participants’ socio-demographic information (i.e., age, sex, immigrant background, school track, parents’ education) was collected at baseline.

#### Identification with the European Group

Identification with the European group was assessed with a shortened version of the Group Identification Scale (Thomas et al., 2017). This version included three items (i.e., “I have a lot in common with other members of the group of Europeans”, “Being a member of the group of Europeans is important to who I am”, “I identify with the group of Europeans”), which adolescents rated on a 5-point Likert type scale from 1 (*completely false*) to 5 (*completely true*). Cronbach’s alphas ranged from 0.79 to 0.89 (see Table S3 of the Supplemental Materials for further details).

#### Daily Identification with the European Group

The extent to which adolescent daily identify with the European group was measured using the single-item “Today I’ve identified with the group of Europeans” (Postmes et al., 2013), which participants rated on a 5-point Likert scale from 1 (*completely false*) to 5 (*completely true*).

### Strategy of Analysis

Descriptive analyses and rank-order stability were conducted using IBM SPSS Version 28.0 for Windows. The remaining analyses were conducted in M*plus* 8.10 (Muthén & Muthén, 2017), using Maximum Likelihood Robust (MLR) estimator (Satorra & Bentler, 2001). Analyses codes and outputs can be retrieved from 10.17605/OSF.IO/43QMS. As a preliminary step, longitudinal and multigroup measurement invariances of identification with the European group were tested (see Supplemental Materials S4). Slightly deviating from the preregistered analytical plan, main analyses were conducted using the *Type* = *Complex* option available in M*plus* providing more robust estimates that account for the nested structure of the data (i.e., students nested within classrooms).

To address the first study goal (i.e., exploring the development of identification with the European group), mean-level changes were examined by conducting Latent Growth Curve (LGC) models for European identification in the medium- (i.e., annual/monthly) and short-term (i.e., daily), separately. Increasingly complex models (i.e., intercept-only, linear, non-linear) were estimated on the total sample and compared against each other to identify the best fitting and most parsimonious solution to represent change (see Supplemental Material S5). Moreover, for each time scale separately, the final model was fitted again in a multigroup format, and Wald tests were used to understand whether growth parameters (i.e., intercept and slope) significantly differed depending on adolescents’ age group. Last, rank-order stability was examined by computing Pearson’s test-retest correlations (i.e., correlations between identification at T1 and T2, T2 and T3, etc.), with coefficients equal or higher than 0.60 indicative of high stability (Mroczek, 2007). Correlation coefficients were subsequently converted using the Fisher *r*-to-*z* transformation to compare them for statistical significance.

Regarding the second goal (i.e., examining the interplay of different time scales), a conditional LGC model was tested to examine whether daily fluctuations in European identification would correlate with its medium-term growth parameters. To this end, a mean fluctuation score was computed by averaging intra-person standard deviations across the three weeks of daily assessments. This model was tested first in the whole sample and then in a multigroup framework.

Regarding the third goal (i.e., explaining variability in developmental trajectories of identification with the European group), a Latent Class Growth Analysis (LCGA) was conducted in several steps. This analytical strategy allows to identify homogeneous subgroups in the population, each characterized by no within-class variance in intercept and slope parameters (Jung & Wickrama, 2008). First, models with an increasing number of classes were tested and compared to identify the best fitting and most parsimonious solution (see Supplemental Materials S6). Next, the selected final class solution was tested again in a multinomial logistic regression model by means of the R3STEP procedure available in M*plus* (Asparouhov & Muthén, 2014) with adolescents’ fluctuation scores and age group (0 = younger, 1 = older) as predictors. The R3STEP command allows to include predictors of the latent class without them affecting the class identification procedure, and therefore to unravel whether these factors would influence the chances of youth being in one developmental trajectory class compared to a reference class. In the current study, the low identification class was selected as reference class.

Last, exploratory (not pre-registered) sensitivity analyses were conducted by including participants’ ethnic background (i.e., majority or minority) as a covariate of short- and medium-term growth parameters (in the LGC models) and their interplay. Additionally, adolescents’ background was included as an additional auxiliary variable of class membership (in the multinomial logistic regression model). Further details are reported in Supplemental Materials S9.

## Results

### Preliminary Analyses

Means, standard deviations, and correlations are reported in Tables S3 and S4 of the Supplemental Materials. Longitudinal and multigroup measurement invariances testing (see Table S5 of the Supplemental Materials) indicated full scalar invariance both across time and groups. Therefore, it was possible to proceed with the main analyses.

### Development of Identification with the European Group

**Fig. 2:**
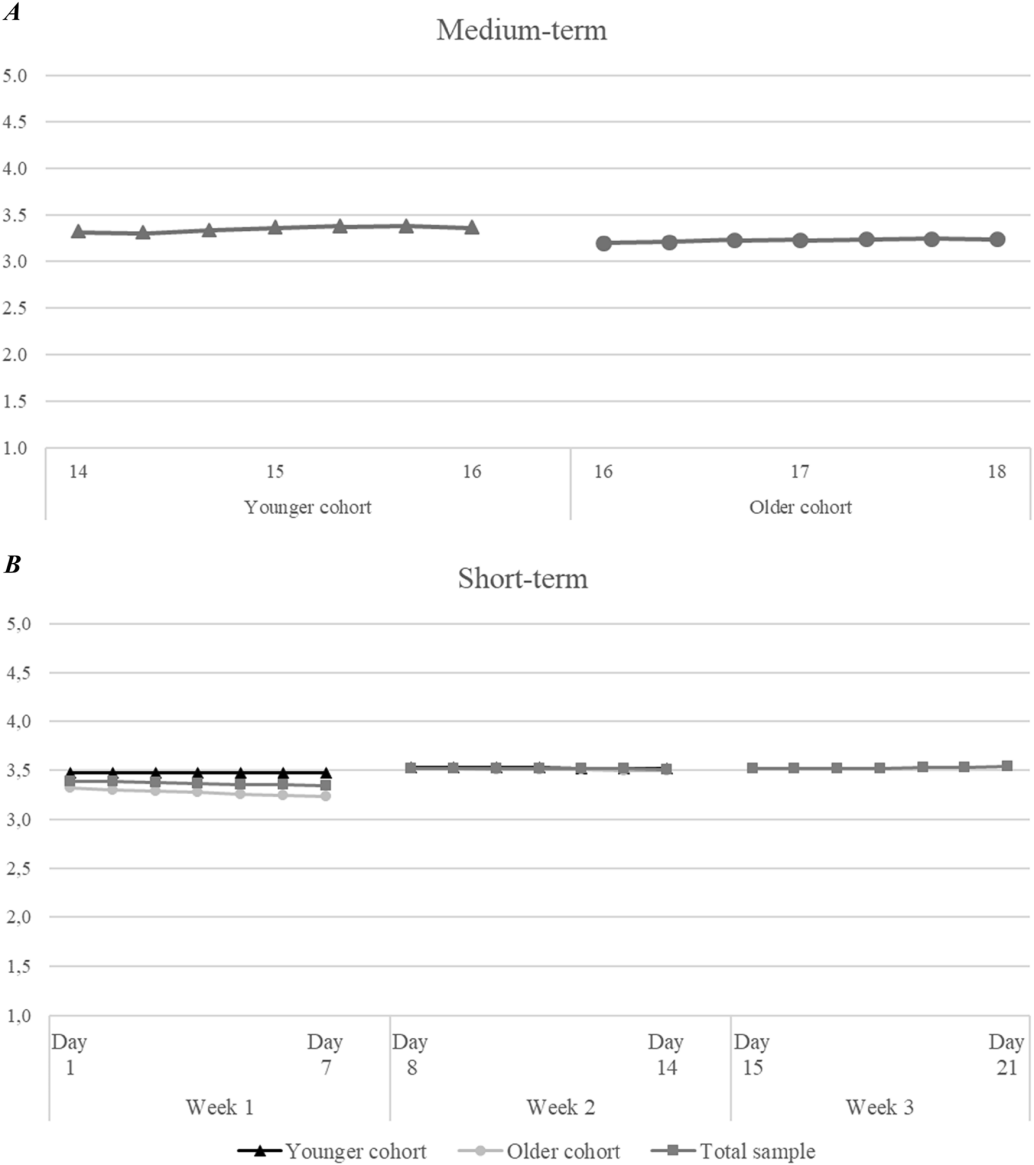
Mean-level changes in identification with the European group. *Note*. Panel **A** displays changes in identification with the European group in the medium-term, separately for younger and older adolescents. Panel **B** displays changes in identification with the European group in the short-term, separately for the total sample and the younger and older cohorts. Mean-level changes in week 2 occurred similarly for the total sample and for both groups. The multigroup version of the Latent Growth Curve model for week 3 did not converge and therefore it was not possible to examine short-term mean-level changes for the younger and older group separately

The first goal of the present study was to examine the development of identification with the European group throughout adolescence, considering both mean-level changes and rank-order stability at the medium- (i.e., annual/monthly) and short-time (i.e., daily) levels. Mean-level changes are reported in Table 1 and represented in Fig. 2 (see also Table S6 for model fit and model selection for the total sample). Rank-order stability coefficients are reported in Table 2.

**Table 1:** Unstandardized growth parameter estimates of Latent Growth Curve models

	Intercept		Slope	
European identification	*M* (SE)	*σ*^*2*^ (SE)	*M* (SE)	*σ*^*2*^ (SE)
	**Medium-term mean-level changes**			
Total sample	3.261^***^ (0.022)	0.269^***^ (0.023)	0.006 (0.003)	0.004^*^ (0.002)
Younger age group	3.315^***^ (0.030)	0.260^***^ (0.032)	0.008^*^ (0.004)	0.002 (0.002)
Older age group	3.196^***^ (0.037)	0.291^***^ (0.058)	0.007 (0.008)	0.007 (0.004)
	**Short-term mean level changes**			
*Week 1*				
Total sample	3.389^***^ (0.048)	0.961^***^ (0.078)	−0.008 (0.007)	0.011^***^ (0.003)
Younger age group	3.468^***^ (0.078)	0.932^***^ (0.099)	0.000 (0.009)	0.011^**^ (0.004)
Older age group	3.318^***^ (0.053)	0.945^***^ (0.115)	−0.015 (0.010)	0.010^**^ (0.003)
*Week 2*				
Total sample	3.522^***^ (0.044)	0.685^***^ (0.058)	−0.002 (0.007)	0.006^**^ (0.002)
Younger age group	3.529^***^ (0.063)	0.655^***^ (0.073)	−0.002 (0.008)	0.004^**^ (0.001)
Older age group	3.519^***^ (0.058)	0.709^***^ (0.086)	−0.004 (0.012)	0.007 (0.004)
*Week 3*^a^				
Total sample	3.514^***^ (0.060)	0.723^***^ (0.096)	0.001 (0.011)	0.009^**^ (0.003)

*M* mean, *SE* standard error, *σ*^*2*^ variance

Bolded values indicate significant differences in the mean scores of younger and older adolescents

^*^ *p* < 0.05; ^**^ *p* < 0.01; ^***^ *p* < 0.001

^a^The multigroup version of the Latent Growth Curve model for week 3 did not converge

**Table 2:** Rank-order stability

European identification	T1-T2	T2-T3	T3-T4	T4-T5	T5-T6	T6-T7
**Medium-term development**						
Total sample	0.449_a_	0.529_b_	0.581_b,c_	0.586_b,c,d_	0.640_d_	0.614_c,d_
Younger age group	0.420_a_	0.545_b_	0.542_b_	0.545_b_	0.600_b_	0.569_b_
Older age group	0.483_a_	0.519_a_	0.618_b_	0.626_b_	0.677_b_	0.669_b_

European identification	D1-D2	D2-D3	D3-D4	D4-D5	D5-D6	D6-D7
**Short-term development**						
*Week 1*						
Total sample	0.653_a_	0.837_b_	0.878_b,d_	0.859_b,c,d_	0.822_b,c_	0.896_d_
Younger age group	0.555_a_	0.849_b_	0.869_c_	0.859_c_	0.773_b_	0.876_c_
Older age group	0.711_a_	0.831_b_	0.892_c,d_	0.860_b_	0.866_b,c_	0.915_d_
*Week 2*						
Total sample	0.676_a_	0.794_b_	0.832_b,c_	0.807_b_	0.875_c,d_	0.899_d_
Younger age group	0.678_a_	0.791_b_	0.872_c,d_	0.830_b,c_	0.925_d_	0.904_d_
Older age group	0.672_a_	0.798_b_	0.784_a,b_	0.782_a,b_	0.823_b_	0.894_c_
*Week 3*						
Total sample	0.786_a_	0.831_a_	0.810_a_	0.804_a_	0.755_a_	0.947_b_
Younger age group	0.751_a,b_	0.773_a,b_	0.716_a_	0.692_a_	0.547_a_	0.883_b_
Older age group	0.827_a_	0.885_a_	0.896_a_	0.910_a_	0.924_a_	1.000_b_

*T* time. *D* day. Subscript letters indicate coefficients within each row that are significantly different from each other. All coefficients were significant at *p* < 0.001

#### Medium-Term Development

Regarding mean-level changes, the multigroup LGC model with a non-linear slope fitted the data very well: χ^2^ = 75.103^***^, CFI = 0.970, TLI = 0.964, RMSEA [90% C.I.] = 0.039 [0.026, 0.051]. Younger adolescents displayed significantly higher identification with the European group (i.e., intercept) compared to older ones (Wald(1) = 6.15, *p* = 0.013). No significant difference (Wald(1) = 0.03, *p* = 0.867) emerged in the slope parameter, which indicated a small, albeit not significant, increase over time for both groups.

Regarding interindividual differences, rank-order stability increased substantially over time in the total sample, as well as within each group. For younger adolescents, the T1-T2 rank-order coefficient was significantly different from all the other coefficients, indicating this as a moment of change in the relative standing of individuals. Conversely, for older youth, the T1-T2 and T2-T3 coefficients were significantly different from the others.

#### Short-Term Development

Regarding mean-level changes, the multigroup LGC model with a linear slope fitted the data well for both the first (χ^2^ = 84.764^***^, CFI = 0.952, TLI = 0.956, RMSEA = 0.058 [0.038, 0.077]) and the second week (χ^2^ = 72.370^**^, CFI = 0.959, TLI = 0.962, RMSEA = 0.047 [0.024, 0.067]) of daily assessment. Across both weeks, initial levels of identification with the European group were high and remained stable for both younger and older adolescents. The multigroup version of the LGC model for the third week of daily collection did not successfully converge, and therefore differences between the two groups of participants could not be examined. However, results on the total sample aligned with the general stability trend observed over the other two weeks of daily assessment, with adolescents displaying high initial levels of identification which remained stable over time. Regarding interindividual differences, rank-order stability was very high for the total sample, as well as for each group. Additionally, significant differences emerged over the days and weeks, indicating a progressively increasing stability in European identification.

### Interplay of Different Time Scales

The second goal of the present study was to understand whether developmental processes occurring in the medium-term are intertwined with daily fluctuations in European identification. Considering the whole sample, adolescents with lower initial levels of identification in the medium-term showed more daily fluctuations (*r* = −0.15, *p* = 0.014). Within a multigroup framework, this negative correlation was confirmed, but only in the younger (*r* = −0.29, *p* = 0.001) and not in the older (*r* = −0.01, *p* = 0.909) group (Wald(1) = 5.53, *p* = 0.019). No significant association emerged between rates of change and daily fluctuations in European identification, both in the whole sample and across groups.

### Variability in Identification with the European Group

**Fig. 3:**
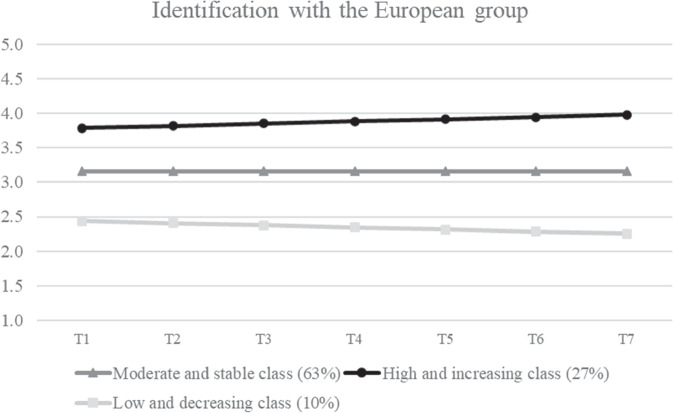
Results of Latent Class Growth Analysis. *Note*. T time

The last goal of the present study was to examine whether variability in youth development in the medium-term could be traced back to the existence of different groups and whether these groups would differ also in terms of their short-term fluctuations. Results of LCGA are reported in Table S7 (model selection) and Table S8 (unstandardized growth parameters) of the Supplemental Materials. A three-class solution was the best fitting one (Fig. 3). The first class, comprising 63% of adolescents, was characterized by moderate levels of identification with the European group, which remained stable over time. The second group included 27% of participants, who displayed high and increasing levels of European identification. Last, the third group, comprising 10% of participants, was characterized by low and decreasing levels of identification with the European group. Wald tests confirmed that the three groups significantly differ in their intercept and slope parameters.

Results of the multinomial logistic regression (Table 3) highlighted that adolescents who fluctuated more in their daily identification levels were also less likely to be in the high compared to the low European identification group. Additionally, older youth were less likely to be in the high compared to the low identification group. This finding was further probed by conducting a chi-square test (χ^2^(2) = 8.987, *p* = 0.011, φ = 0.078) on the distribution of younger and older adolescents within the three developmental classes. The low European identification group included a lower number of younger (42.25%) compared to older (57.75%) adolescents, whereas the high European identification group comprised a higher percentage of younger (56.67%) rather than older (43.33%) participants. Daily fluctuations and adolescents’ age group did not significantly influence the chances of participants being in the average compared to the low identification group.

**Table 3:** Multinomial logistic regression

	Average identification group vs. Low identification group		High identification group vs. Low identification group	
Predictors	*B* (*SE*)	OR [95% C.I.]	*B* (*SE*)	OR [95% C.I.]
Daily fluctuations	−0.934 (0.534)	0.393 [0.138, 1.120]	−1.797^**^ (0.538)	0.166 [0.058, 0.477]
Age group	−0.340 (0.319)	0.712 [0.381, 1.331]	−0.632^*^ (0.321)	0.531 [0.283, 0.997]

Age group: *0* younger, *1* older. *B* Unstandardized regression parameter, *SE* standard error, *OR* odds ratio, *CI* confidence interval

^*^ *p* < 0.05; ^**^ *p* < 0.01

### Sensitivity Analyses

Sensitivity analyses controlling for participants’ ethnic background fully replicated the main findings. Medium- and short-term developmental patterns as well as their interplay were similar for ethnic minority and majority participants. Only one significant association between adolescents’ ethnic background and European identification in the medium-term emerged, and only for the younger cohort. Younger ethnic majority youth displayed slightly higher initial levels compared to younger ethnic minority youth (see Table S9). Additionally, when controlling participants’ ethnic background, the age cohort was only marginally (*p* = 0.051) significantly associated with the chances of being in the high compared to the low identification group (see Table S10).

## Discussion

Identification with the European group is a relevant social membership for adolescents living in European countries (Landberg et al., 2018), and it bears important implications for their civic participation and intergroup attitudes (e.g., Bobba et al., 2024b). This study examined the developmental trajectories of identification with the European group among younger and older Italian adolescents across short- and medium-term timescales, separately and conjointly. Further, it focused on heterogeneity in medium-term developmental patterns and whether participants’ age and short-term fluctuations would explain such variability. The findings point to general stability in identification with the European group in both the short- and medium-term, and highlight significant differences between younger and older adolescents. Moreover, three groups of adolescents were found characterized by low, medium, and high (initial levels of) European identification, and both age and daily fluctuations explained membership in these different developmental trajectory groups. All in all, this study extends knowledge on identity formation by shedding light on how the development of European identification unfolds across multiple time scales and age groups.

### Social Identification with the European Group Across Time

The study’s first aim was to examine how identification with the European group changes in the medium- (i.e., monthly/yearly) and in the short-term (i.e., daily) considering adolescents’ age group. First, changes of mean-levels in identification with the European group were examined. Then, rank-order stability was examined.

#### Mean-Level Changes

In line with Hypothesis 1, identification with the European group remained mostly stable over time across both time scales. Additionally, in the medium-term, older adolescents reported significantly lower initial levels of identification with the European group compared to their younger peers, while no differences across the two age groups emerged in the short-term. The results are in line with previous research examining development of personal identity (Becht et al., 2021).

When examining the medium-term timescale more closely, it was found that identification with the European group increased slightly for both groups, albeit not significantly. This means that there might be a general trend towards stronger identification with the European group over time (i.e., signifying a maturation process), but across a three-year period identification with the European group can be considered stable. This is consistent with results from personal identity (for a review see Branje et al., 2021). Identity development has been characterized by little mean-level changes in identity processes from adolescence to young adulthood irrespective of examined identity domain (e.g., cultural, Kranz & Goedderz, 2020; regional, Schubach et al., 2017). However, the lack of significant developmental change contrasts with comparable findings on European identity (Mayer et al., 2024). Previous studies found a significant increase followed by a decrease in all European identity processes across a school year. It was argued that these significant changes might have been caused by specific contextual events (i.e., Russia’s invasion of Ukraine and learning about the EU and Europe in school), occurring in the middle of their observation period. Notably, Russia’s invasion of Ukraine also happened at the beginning of the current observation period and therefore could have systematically influenced the initial levels of identification with the European group as well. While initial level of identification might have been affected, later time points were likely unaffected (see the return to initial levels of identity processes from mid to end school year in Mayer et al., 2024). Thus, the development of European identification might have followed more general patterns of identity development, similar to national and ethnic identification (see, for example, Karataş et al., 2023).

Relating to the potential influence of contextual factors, lower initial levels of identification with the European group were found in older compared to younger adolescents. If identification with the European group were to follow general trends in identity development, both groups should not have differed, or older adolescents should have shown higher initial levels. One explanation might be that older adolescents were more negatively affected by averse contextual factors (i.e., Russia’s invasion of Ukraine), because they were more aware of those events due to maturation processes (i.e., cognitive capacity to understand distal events). However, it is important to keep in mind that differences in initial levels were small (older: *M* = 3.20 versus younger: *M* = 3.32). Future studies on identity development could include contextual factors to control for their effects when studying social identities, especially if they are potentially political.

#### Rank-Order Stability

In line with Hypothesis 2, high and increasing rank-order stability over time on both time scales was found, meaning that participants relative position within the sample became more stable over time. These findings are in line with previous research on personal identity (for a review see Meeus, 2011) and European identity (Mayer et al., 2024), and is comparable to developmental trends of personality traits (e.g., Bleidorn, et al., 2022; see Meeus, 2019 for review). Rank-order stability was higher on the short-term than on the medium-term time scale, which can be explained by the far shorter time lag between measurements. Stability is inversely related to the time lag between assessments (Crocetti et al., 2021), resulting in higher stabilities across days than months. Furthermore, rank-order stability increased from the first to the last measurement occasion across each short-term period.

Regarding the medium-term time scale, younger adolescents showed a significant increase in rank-order stability between T2-T3 (i.e., around age 14). For older adolescents, the significant increase in rank-order stability was observed between T3-T4 (i.e., around 17), which was again followed by stability. Taken together, the results could indicate that around 14 and 17 youth progressively stabilize their European identification relative to their peers. The change point in interindividual stability around 14 might be linked to cognitive maturation processes during puberty, as it is a crucial period for the development and consolidation of personal views about self, others, and society (Meeus, 2019). The change point in interindividual stability around 17 might be linked to adolescents’ engagement in Italian national elections. Since the legal voting age in Italy is 18, the older group might have engaged more thoroughly with EU- and Europe-related topics since they were approaching their first political vote. In the national political elections held at the end of September 2022, those topics were among the most discussed for differentiating voting positions (e.g., right-wing and anti-EU versus left-wing and pro-EU). The heightened engagement might have led to a boost in consolidation of personal positions.

### Interrelation of Short- and Medium-Term Timescales

The study’s second aim was to unravel the developmental interplay between medium-term changes and daily fluctuations. Partially in line with Hypothesis 3, participants with higher initial levels of identification with the European group fluctuated less during each of the three weeks of daily observations. The lower levels of fluctuations could be indicative of a firm sense of self with fewer evaluations of definitions of group membership (Luyckx et al., 2006). In other words, participants who identify highly with the European group would not question their group belonging on a daily level. An association between a firm sense of self and long-term stability has also been found in studies on personal identity (for educational and interpersonal identity; Becht et al., 2021).

It is noteworthy that the negative correlation between initial levels and daily fluctuations of identification with the European group was only significant in the younger and not in the older age group. It could be that younger adolescents are in the process of figuring out whether they identify with the European group or not. Older adolescents might have already securely decided to do so or not. Both groups might have been on different developmental stages of European identification, which aligns with research on personal identity (see Branje et al., 2021). Identity can be assumed to develop through three interacting dynamic processes: commitment, in-depth exploration, and reconsideration of commitment (Crocetti et al., 2008). Depending on adolescents’ levels in those three processes, one can infer how they deal with the task of identity formation (e.g., achieved identity: high levels of commitment and in-depth exploration, low levels of reconsideration). Future studies could include those processes to additionally capture adolescents’ developmental differences also in identification with social groups.

Refuting Hypothesis 4, no significant association between change across time and daily fluctuations in European identification was found. It was expected that high levels of fluctuation would be associated with changes in identification with the European group (e.g., Becht et al., 2016), which could have resulted in either increasing or decreasing levels of European identification. However, adolescents reported generally stable levels of identification with the European group. This suggests that daily fluctuations might have little implications for changes occurring in the medium-term.

### Identifying Groups of Adolescents Showing Differences in European Identity

The third aim of the study was to examine whether variability in medium-term mean-level changes could be traced back to the existence of different groups of participants. Further, it aimed to investigate whether adolescents’ age and daily fluctuations could be associated with unique developmental trajectories of identification with the European group. In line with both Hypotheses 5 and 6, three trajectory groups of identification with the European group that differed in their levels of identification and strength of daily fluctuations were found. Almost two-thirds of the participants were in a group with moderate and stable levels of identification with the European group, around a quarter were in a group with high and increasing levels, and the remaining were in a group with low and decreasing levels. Results indicated that, similar to personal identity, most adolescents do not change much in their identification with the European group (Branje et al., 2021). Nevertheless, if they do, it is more often in the direction of increasing identification, which could imply an ongoing process of identity maturation.

Participants in the moderate group did not differ in their daily fluctuations compared to both other groups. Conversely, the group with high and increasing levels fluctuated significantly less compared to the group with low and decreasing levels of identification with the European group. In line with the results above, higher levels of identification might indicate a firm and stable sense of identification, which is accompanied by little or no re-evaluation of group membership on a daily level. However, if adolescents are unsure about their identification, they are also more likely to re-evaluate their identifications and explore different meanings.

Interestingly, older adolescents were more likely in the low and decreasing identification group than in the high and increasing group compared to younger adolescents. Older adolescents could have been more aware of political events on the European level due to cognitive maturation processes (Erikson, 1968) and might have therefore reconsidered whether they wanted to belong to the European group more than younger adolescents. Alternatively, these findings might be explained in relation to decreases in social trust, which were found to occur throughout adolescence (Flanagan & Stout, 2010). In other words, older youth might believe less in the other people’s fairness and trustworthiness, which might lead them to evaluate the European group and its members more critically and thus identify to a lesser extent with them.

### Limitations and Future Directions

The current study has several strengths, including the time span considered, the focus on both short- and medium-term developmental trajectories, and the comparison of two age groups of younger and older adolescents. However, its findings should be considered in light of two limitations. First, with the current observation period, it is difficult to disentangle age versus cohort-related effects. Adolescents’ identification with the European group might decrease across adolescence before it increases again, or the older age group might differ systematically from the younger age group (e.g., higher awareness of Russia’s invasion of Ukraine at the beginning of the study period). Future studies could adopt a longer observation period, including the same individuals, to cover the full course of adolescence.

Second, differences between different dynamic processes sustaining identity development could not be examined. This means that the weakening and strengthening in identification with the European group could be observed but whether this is caused by reconsideration of those identifications, which might lead to changes in identification, or whether it is part of an ongoing in-depth exploration process that does not question the identification per se, could not be examined. Future studies could assess identity processes to examine their interplay with social identifications (Crocetti et al., 2018).

Another limitation of the present study lies in its exclusive focus on European identification. Adolescents construct their identity across multiple domains, and the relevance of these domains is likely to vary throughout adolescence and across the life span (e.g., de Moor & Olaru, 2025; Vosylis et al., 2018). In particular, the salience of group identifications—such as national, ethnic, or supranational identities—may fluctuate over time, depending on both developmental phases and contextual influences. To tackle this issue, future research should adopt a developmental perspective to better understand how the importance of multiple identity domains, including group-based identities, evolves during adolescence.

### Practical Implications

This study can have important practical implications and contribute to defining interventions (Crocetti et al., 2025; Piotrowski et al., 2025) and educational practices to enhance European identification. Considering the difference in development, if educators wish to promote European identification, they should implement stage-adequate strategies. For younger adolescents, this could mean to aid formation and consolidation of European identification. This could be established by providing relevant information about Europe and Europeans, and promoting intercultural exchange (e.g., student exchange, European weeks). For older adolescents, this could mean strengthening identification, media literacy, and efficacy regarding political events, and counteracting the decrease in social trust. This could be established by providing strategies on critically engaging in news and, again, promoting intercultural exchange. Throughout adolescence, educators will have to take care of the criteria for being European they teach. The European identity has been found to be a unifying social identity relevant for ethnically diverse Europeans, but only if it is inclusively and civically defined. Combining social and developmental approaches, this study provides a comprehensive understanding of how adolescents form and consolidate their identification with the European group, a relevant resource promoting youth’s adjustment and participation to current multicultural societies.

## Conclusion

Forming and consolidating a sense of identification with relevant social groups, such as the European one, is a crucial developmental task in adolescence and has important implications for youth socio-political adjustment. This longitudinal study sheds light on how younger and older adolescents develop their identification with the European group in the short- and medium-term, on the interplay of these different timescales, and on heterogeneity in medium-term developmental trajectories. These findings highlight that, despite general stability in the short- and medium-term, younger and older adolescents might be at different stages in their development of identification with the European group. Moreover, adolescents’ age and fluctuations in the short-term were linked to unique medium-term developmental trajectories of identification.

## Supplementary Information

Supplemental materials_FINAL
