# Supplementary material for: Young Europeans – The Interplay between Short- and Medium-Term Development of European Identification Across Adolescence
Source: J Youth Adolesc. 2025 Sep 22;55(1):196–210. doi: 10.1007/s10964-025-02256-y (PMC12816093; doi:10.1007/s10964-025-02256-y)
Supplement: Supplementary file 1 — Supplemental materials_FINAL [file 10964_2025_2256_MOESM1_ESM.docx]

**SUPPLEMENTAL MATERIALS**

**“ Young Europeans – The Interplay Between Short- and Medium-Term Development of European Identification Across Adolescence”**

| **S1. Participants’ ethnic background and group comparisons** | 2 |
| --- | --- |
| **S2. Sample attrition analyses** | 4 |
| **Table S3.** Means, standard deviations, and reliability of study variables | 5 |
| **Table S4.** Correlations among study variables | 6 |
| **S5. Longitudinal and multigroup measurement invariance** | 8 |
| **Table S5.** Measurement invariance of European identification | 9 |
| **S6. Latent Growth Curve Models: Model selection and results in the whole sample** | 10 |
| **Table S6.** Latent Growth Curve Models: Model comparison | 11 |
| **S7. Latent Class Growth Analysis: Model selection procedure and results** | 12 |
| **Table S7.** Latent Class Growth Analysis: Model comparison | 13 |
| **Table S8.** Unstandardized growth parameters of the LCGA three-class solution | 14 |
| **S9. Sensitivity analyses** | 15 |
| **Table S9.** Latent Growth Curve Models with covariate: Model fit and standardized correlations | 16 |
| **Table S10.** Multinomial logistic regression with covariates | 17 |

**S1. Participants’ Ethnic Background and Group Comparisons**

The current study examined the development of European identification in a sample of adolescents from the Italian general population. Ethnic majority youth whose parents were both born in Italy accounted for 79.87% of the total sample (*M*_age_=15.63, *SD*_age_=1.17; 46.09% females; 52.67% 1^st^ year students), while the remaining 20.13% were ethnic minority adolescents with at least one parent born outside Italy (*M*_age_=15.96, *SD*_age_=1.38; 50.81% females; 50.81% 1^st^ year students). Of these, 26.24% were born abroad (i.e., first-generation). Among first-generation immigrant youth, most were born in Eastern European (e.g., Albania; 36.76%), Asian (e.g., Pakistan; 29.40%) or African (e.g., Nigeria; 24.99%) countries, while a few were born in other Central European countries (e.g., Germany; 4.41%) or in South (e.g., Brazil; 2.94%) and North (i.e., USA; 1.50%) America. In a similar way, parents of second-generation immigrant adolescents were born either in Eastern European (39.15% of mothers and 29.20% of fathers), African (29.80% of mothers and 40.01% of fathers), or Asian (12.26% of mothers and 15.39% of fathers) countries. The remaining were born in other Central European countries (7.13% of mothers and 9.24% of fathers), in Central/South (9.92% of mothers and 6.16% of fathers) and North (1.16% of mothers) America, or Australia (0.58% of mothers).

Adolescents in the two groups did not differ in terms of sex (χ^2^(df) = 2.199(1), *p* = .142; φ = .04) and age cohort (χ^2^(df) = 0.345(1), *p* = .567; Kendall τ = .01), although ethnic minority youth were slightly older on average compared to their ethnic majority peers (*t*(df) = -4.078(1,425), *p* < .001; Cohen’s *d*[95% C.I.] =

-.27[-.40, -.14]). Furthermore, ethnic majority and minority adolescents significantly differed in their mothers’ (χ^2^(df) = 37.919(2), *p* < .001; η = .15) and fathers’ (χ^2^(df) = 13.293(2), *p* = .001; η = .09) educational level. Specifically, mothers of ethnic minority adolescents were more likely to have a low (34.58%) and less likely to have a high (23.33%) educational level compared to mothers of ethnic majority youth (17.23% with low and 34.00% with high educational level). Similarly, fathers of ethnic minority participants were more represented in the low (37.76%) and less represented in the high (18.67%) educational levels, while for ethnic majority youth the distribution across the two educational levels was more even (26.45% of fathers in the low and 25.16% in the high educational level).

Considering monthly/annual assessments, ethnic majority and minority adolescents did not significantly differ in their levels of identification with the European group at T1 (*t*(df) = 0.534(1,044), *p* = .594), T2 (*t*(df) = -1.257(1,018), *p* = .209), T3 (*t*(df) = -1.926(874), *p* = .054), T4 (*t*(df) = -0.994(863), *p* = .320), T5 (*t*(df) = -1.897(815), *p* = .058), T6 (*t*(df) = -0.480(760), *p* = .631), and T7 (*t*(df) = -0.894(759), *p* = .372). Similarly, no differences emerged in mean scores of European identification throughout each week of daily assessments (first week: Day 1: *t*(df) = -0.142(342), *p* = .888; Day 2: *t*(df) = 0.491(339), *p* = .623; Day 3: *t*(df) = 0.616(299), *p* = .539, Day 4: *t*(df) = 0.162(291), *p* = .872, Day 5: *t*(df) = 0.496(272), *p* = .621; Day 6: *t*(df) = 0.647(262), *p* = .518; Day 7: *t*(df) = -0.070(252), *p* = .945; second week: Day 1: *t*(df) = 0.080(369), *p* = .936; Day 2: *t*(df) = -0.002(314), *p* = .998; Day 3: *t*(df) = 0.495(286), *p* = .621, Day 4: *t*(df) = 0.246(267), *p* = .806, Day 5: *t*(df) = 0.394(263), *p* = .694; Day 6: *t*(df) = 0.283(234), *p* = .777; Day 7: *t*(df) = 0.925(245), *p* = .356; third week: Day 1: *t*(df) = 0.150(175), *p* = .881; Day 2: *t*(df) = 1.092(146), *p* = .277; Day 3: *t*(df) = 1.148(123), *p* = .253, Day 4: *t*(df) = 0.765(114), *p* = .446, Day 5: *t*(df) = 1.552(100), *p* = .124; Day 6: *t*(df) = 1.790(80), *p* = .077; Day 7: *t*(df) = 0.742(84), *p* = .460).

**S2. Sample Attritions Analyses**

The current study included multiple annual/monthly assessments (during school hours) and daily evaluations (online). A total of 1,552 adolescents were included in the current study. Of these, approximately half (50.13%) participated only in the annual/monthly assessments, almost all the remaining (49.55%) completed both annual/monthly and daily assessments, and a few (0.32%) completed only the daily questionnaires.

Among adolescents participating in the annual/monthly assessments, most (35.22%) completed more than half of the evaluations (i.e., between four and six), 24.37% completed all seven of them, while 23.22% participated for less than half of the assessments (i.e., between two and three), and 17.19% participated only once. Within each assessment, the completion rate ranged from 67.23% at T1 to 48.93% at T7, and missingness was mostly due to participants not filling out the questionnaire because they were not in school on the day of data collection. Among adolescents participating in the daily evaluations, most of them (48.45%) completed only one, followed by those who completed two (29.59%), and those who completed three (21.96%) week of daily assessments.

**Table S3**

Means (*M*), standard deviations (*SD*), and reliability (α) of study variables

| Identification with Europeans | Monthly assessments | | | Daily assessments | | | | | |
| --- | --- | --- | --- | --- | --- | --- | --- | --- | --- |
|  |  | | | Week 1 | | Week 2 | | Week 3 | |
|  | *M* | *SD* | α | *M* | *SD* | *M* | *SD* | *M* | *SD* |
| Time 1 | 3.27 | 0.74 | .79 |  |  |  |  |  |  |
| Time 2 | 3.31 | 0.76 | .81 |  |  |  |  |  |  |
| Time 3 | 3.29 | 0.74 | .84 |  |  |  |  |  |  |
| Time 4 | 3.29 | 0.80 | .87 |  |  |  |  |  |  |
| Time 5 | 3.35 | 0.77 | .85 |  |  |  |  |  |  |
| Time 6 | 3.36 | 0.79 | .89 |  |  |  |  |  |  |
| Time 7 | 3.34 | 0.76 | .87 |  |  |  |  |  |  |
|  |  |  |  |  |  |  |  |  |  |
| Day 1 |  |  |  | 3.45 | 1.06 | 3.60 | 0.97 | 3.53 | 0.92 |
| Day 2 |  |  |  | 3.38 | 1.12 | 3.47 | 1.00 | 3.56 | 0.99 |
| Day 3 |  |  |  | 3.40 | 1.08 | 3.50 | 1.03 | 3.57 | 1.01 |
| Day 4 |  |  |  | 3.41 | 1.04 | 3.51 | 0.99 | 3.66 | 1.06 |
| Day 5 |  |  |  | 3.40 | 1.08 | 3.46 | 1.03 | 3.47 | 1.05 |
| Day 6 |  |  |  | 3.45 | 1.09 | 3.55 | 1.00 | 3.57 | 1.07 |
| Day 7 |  |  |  | 3.39 | 1.09 | 3.51 | 1.00 | 3.49 | 1.13 |

**Table S4**

Correlations among study variables

|  | 1. | 2. | 3. | 4. | 5. | 6. | 7. | 8. | 9. | 10. | 11. | 12. | 13. | 14. |
| --- | --- | --- | --- | --- | --- | --- | --- | --- | --- | --- | --- | --- | --- | --- |
| 1.Identification with Europeans T1 |  |  |  |  |  |  |  |  |  |  |  |  |  |  |
| 2.Identification with Europeans T2 | .45^***^ |  |  |  |  |  |  |  |  |  |  |  |  |  |
| 3.Identification with Europeans T3 | .46^***^ | .53^***^ |  |  |  |  |  |  |  |  |  |  |  |  |
| 4.Identification with Europeans T4 | .42^***^ | .45^***^ | .58^***^ |  |  |  |  |  |  |  |  |  |  |  |
| 5.Identification with Europeans T5 | .39^***^ | .46^***^ | .59^***^ | .59^***^ |  |  |  |  |  |  |  |  |  |  |
| 6.Identification with Europeans T6 | .40^***^ | .41^***^ | .59^***^ | .60^***^ | .64^***^ |  |  |  |  |  |  |  |  |  |
| 7.Identification with Europeans T7 | .40^***^ | .37^***^ | .56^***^ | .53^***^ | .56^***^ | .61^***^ |  |  |  |  |  |  |  |  |
| 8.Identification with Europeans D1 | .37^***^ | .35^***^ | .33^***^ | .28^***^ | .36^***^ | .25^***^ | .32^***^ |  |  |  |  |  |  |  |
| 9.Identification with Europeans D2 | .34^***^ | .29^***^ | .39^***^ | .38^***^ | .46^***^ | .31^***^ | .29^***^ | .65^***^ |  |  |  |  |  |  |
| 10.Identification with Europeans D3 | .41^***^ | .35^***^ | .44^***^ | .43^***^ | .57^***^ | .37^***^ | .37^***^ | .66^***^ | .84^***^ |  |  |  |  |  |
| 11.Identification with Europeans D4 | .42^***^ | .39^***^ | .42^***^ | .46^***^ | .42^***^ | .33^***^ | .32^***^ | .72^***^ | .82^***^ | .88^***^ |  |  |  |  |
| 12.Identification with Europeans D5 | .38^***^ | .36^***^ | .55^***^ | .54^***^ | .52^***^ | .42^***^ | .40^***^ | .64^***^ | .79^***^ | .85^***^ | .86^***^ |  |  |  |
| 13.Identification with Europeans D6 | .34^***^ | .34^***^ | .50^***^ | .55^***^ | .48^***^ | .31^***^ | .33^***^ | .66^***^ | .79^***^ | .79^***^ | .80^***^ | .82^***^ |  |  |
| 14.Identification with Europeans D7 | .33^***^ | .41^***^ | .51^***^ | .57^***^ | .57^***^ | .39^***^ | .41^***^ | .58^***^ | .71^***^ | .77^***^ | .76^***^ | .80^***^ | .90^***^ |  |
| 15.Identification with Europeans D8 | .37^***^ | .44^***^ | .54^***^ | .59^***^ | .57^***^ | .52^***^ | .37^***^ | .47^***^ | .50^***^ | .51^***^ | .49^***^ | .59^***^ | .55^***^ | .50^***^ |
| 16.Identification with Europeans D9 | .36^***^ | .32^***^ | .45^***^ | .59^***^ | .47^***^ | .54^***^ | .35^***^ | .44^***^ | .46^***^ | .45^***^ | .42^***^ | .48^***^ | .60^***^ | .53^***^ |
| 17.Identification with Europeans D10 | .43^***^ | .39^***^ | .60^***^ | .63^***^ | .54^***^ | .53^***^ | .39^***^ | .39^***^ | .49^***^ | .53^***^ | .48^***^ | .57^***^ | .61^***^ | .52^***^ |
| 18.Identification with Europeans D11 | .36^***^ | .38^***^ | .57^***^ | .67^***^ | .59^***^ | .55^***^ | .33^***^ | .39^***^ | .43^***^ | .40^***^ | .45^***^ | .52^***^ | .61^***^ | .60^***^ |
| 19.Identification with Europeans D12 | .32^***^ | .40^***^ | .59^***^ | .69^***^ | .63^***^ | .62^***^ | .40^***^ | .42^***^ | .50^***^ | .47^***^ | .48^***^ | .56^***^ | .60^***^ | .61^***^ |
| 20.Identification with Europeans D13 | .37^***^ | .40^***^ | .58^***^ | .67^***^ | .61^***^ | .56^***^ | .37^***^ | .41^***^ | .51^***^ | .51^***^ | .50^***^ | .58^***^ | .64^***^ | .61^***^ |
| 21.Identification with Europeans D14 | .29^***^ | .40^***^ | .50^***^ | .63^***^ | .55^***^ | .55^***^ | .40^***^ | .41^***^ | .48^***^ | .48^***^ | .48^***^ | .57^***^ | .58^***^ | .56^***^ |
| 22.Identification with Europeans D15 | .31^***^ | .26^**^ | .54^***^ | .54^***^ | .59^***^ | .57^***^ | .52^***^ | .29^**^ | .40^***^ | .38^***^ | .42^***^ | .44^***^ | .43^***^ | .45^***^ |
| 23.Identification with Europeans D16 | .39^***^ | .29^**^ | .58^***^ | .62^***^ | .57^***^ | .62^***^ | .46^***^ | .29^**^ | .37^***^ | .40^***^ | .41^***^ | .46^***^ | .54^***^ | .52^***^ |
| 24.Identification with Europeans D17 | .38^***^ | .39^***^ | .58^***^ | .68^***^ | .57^***^ | .68^***^ | .52^***^ | .28^**^ | .38^***^ | .46^***^ | .41^***^ | .49^***^ | .67^***^ | .62^***^ |
| 25.Identification with Europeans D18 | .40^***^ | .36^***^ | .51^***^ | .54^***^ | .57^***^ | .64^***^ | .47^***^ | .30^*^ | .45^***^ | .50^***^ | .55^***^ | .58^***^ | .62^***^ | .52^***^ |
| 26.Identification with Europeans D19 | .39^**^ | .35^***^ | .43^***^ | .66^***^ | .52^***^ | .63^***^ | .47^***^ | .35^**^ | .44^***^ | .50^***^ | .59^***^ | .63^***^ | .58^***^ | .55^***^ |
| 27.Identification with Europeans D20 | .35^***^ | .54^***^ | .64^***^ | .70^***^ | .68^***^ | .79^***^ | .58^***^ | .39^**^ | .36^**^ | .35^**^ | .44^***^ | .49^***^ | .54^***^ | .46^***^ |
| 28.Identification with Europeans D21 | .42^***^ | .37^***^ | .68^***^ | .75^***^ | .77^***^ | .77^***^ | .57^***^ | .43^**^ | .41^**^ | .36^**^ | .54^***^ | .57^***^ | .69^***^ | .70^***^ |

(continued on the following page)

**Table S4 (continued)**

|  | 15. | 16. | 17. | 18. | 19. | 20. | 21. | 22. | 23. | 24. | 25. | 26. | 27. |
| --- | --- | --- | --- | --- | --- | --- | --- | --- | --- | --- | --- | --- | --- |
| 1.Identification with Europeans T1 |  |  |  |  |  |  |  |  |  |  |  |  |  |
| 2.Identification with Europeans T2 |  |  |  |  |  |  |  |  |  |  |  |  |  |
| 3.Identification with Europeans T3 |  |  |  |  |  |  |  |  |  |  |  |  |  |
| 4.Identification with Europeans T4 |  |  |  |  |  |  |  |  |  |  |  |  |  |
| 5.Identification with Europeans T5 |  |  |  |  |  |  |  |  |  |  |  |  |  |
| 6.Identification with Europeans T6 |  |  |  |  |  |  |  |  |  |  |  |  |  |
| 7.Identification with Europeans T7 |  |  |  |  |  |  |  |  |  |  |  |  |  |
| 8.Identification with Europeans D1 |  |  |  |  |  |  |  |  |  |  |  |  |  |
| 9.Identification with Europeans D2 |  |  |  |  |  |  |  |  |  |  |  |  |  |
| 10.Identification with Europeans D3 |  |  |  |  |  |  |  |  |  |  |  |  |  |
| 11.Identification with Europeans D4 |  |  |  |  |  |  |  |  |  |  |  |  |  |
| 12.Identification with Europeans D5 |  |  |  |  |  |  |  |  |  |  |  |  |  |
| 13.Identification with Europeans D6 |  |  |  |  |  |  |  |  |  |  |  |  |  |
| 14.Identification with Europeans D7 |  |  |  |  |  |  |  |  |  |  |  |  |  |
| 15.Identification with Europeans D8 |  |  |  |  |  |  |  |  |  |  |  |  |  |
| 16.Identification with Europeans D9 | .68^***^ |  |  |  |  |  |  |  |  |  |  |  |  |
| 17.Identification with Europeans D10 | .63^***^ | .79^***^ |  |  |  |  |  |  |  |  |  |  |  |
| 18.Identification with Europeans D11 | .69^***^ | .80^***^ | .83^***^ |  |  |  |  |  |  |  |  |  |  |
| 19.Identification with Europeans D12 | .69^***^ | .78^***^ | .80^***^ | .80^***^ |  |  |  |  |  |  |  |  |  |
| 20.Identification with Europeans D13 | .69^***^ | .76^***^ | .86^***^ | .80^***^ | .87^***^ |  |  |  |  |  |  |  |  |
| 21.Identification with Europeans D14 | .62^***^ | .73^***^ | .85^***^ | .75^***^ | .86^***^ | .90^***^ |  |  |  |  |  |  |  |
| 22.Identification with Europeans D15 | .57^***^ | .61^***^ | .53^***^ | .59^***^ | .64^***^ | .70^***^ | .60^***^ |  |  |  |  |  |  |
| 23.Identification with Europeans D16 | .54^***^ | .63^***^ | .64^***^ | .66^***^ | .66^***^ | .66^***^ | .61^***^ | .79^***^ |  |  |  |  |  |
| 24.Identification with Europeans D17 | .58^***^ | .77^***^ | .71^***^ | .66^***^ | .63^***^ | .70^***^ | .60^***^ | .68^***^ | .83^***^ |  |  |  |  |
| 25.Identification with Europeans D18 | .62^***^ | .69^***^ | .63^***^ | .57^***^ | .67^***^ | .77^***^ | .71^***^ | .72^***^ | .76^***^ | .81^***^ |  |  |  |
| 26.Identification with Europeans D19 | .51^***^ | .57^***^ | .68^***^ | .53^***^ | .67^***^ | .66^***^ | .68^***^ | .72^***^ | .81^***^ | .79^***^ | .80^***^ |  |  |
| 27.Identification with Europeans D20 | .58^***^ | .69^***^ | .61^***^ | .55^***^ | .64^***^ | .69^***^ | .71^***^ | .71^***^ | .75^***^ | .77^***^ | .89^***^ | .75^***^ |  |
| 28.Identification with Europeans D21 | .65^***^ | .77^***^ | .62^***^ | .73^***^ | .73^***^ | .72^***^ | .66^***^ | .83^***^ | .88^***^ | .89^***^ | .92^***^ | .91^***^ | .95^***^ |

*Note.* D1 – D7 refer to the first week of daily assessments; D8 – D14 refer to the second week of daily assessment; D15 – D20 refer to the third week of daily assessment.

^*^ *p* < 0.05; ^**^ *p* < 0.01; ^***^ *p* < 0.001.

**S5. Longitudinal and multigroup measurement invariance**

As a preliminary step, configural, metric, and scalar levels of measurement invariance were tested for identification with the European group, both longitudinally (i.e., invariance across the seven monthly assessments) and in a multigroup framework (i.e., invariance across participants of the two cohorts). To this end, the configural models are first estimated as baseline models and their fit evaluated based on the following criteria. The Comparative Fit Index (CFI) and the Tucker–Lewis Index (TLI) with values higher than .90 and .95 indicate an acceptable and very good fit, respectively. The Root Mean Square Error of Approximation (RMSEA) with values below .08 and .05 are indicative of an acceptable and very good fit, respectively (Byrne, 2012), and the RMSEA’s 90% confidence interval’s upper bound lower than .10 indicates an acceptable fit of the model (Chen et al., 2008). In order to establish metric (i.e., constraining factor loadings to be equal across time/groups) and scalar (i.e., constraining intercepts to be equal across time/groups) invariances, changes in fit indices from the configural to the metric model and from the metric model to the scalar were evaluated (e.g., Cheung & Rensvold, 2002). Specifically, a significant Δχ_SB_^2^ (Satorra & Bentler, 2001), and ΔCFI ≥ -.010 supplemented by ΔRMSEA ≥ .015 (Chen, 2007) are indicative of non-invariance. Results are displayed in Table S5. As can be inferred, full scalar invariance was reached both longitudinally and across groups.

**Table S5**

Measurement invariance of European identification

| Models | Model fit | | | | |  |  | Model comparisons | | |
| --- | --- | --- | --- | --- | --- | --- | --- | --- | --- | --- |
|  | χ^2^ | df | CFI | TLI | RMSEA  [90% CI] |  | Models | Δχ_SB_^2^ | ΔCFI | ΔRMSEA |
| Longitudinal measurement invariance | | | | | | | | | | |
| Configural (M1) | 94.425 | 105 | 1.000 | 1.000 | .000  [.000, .010] |  |  |  |  |  |
| Metric (M2) | 118.246 | 117 | 1.000 | 1.000 | .003  [.000, .013] |  | M2-M1 | 27.600 (12)^**^ | .000 | .003 |
| Scalar (M3) | 179.107 | 135 | .994 | .990 | .015  [.008, .020] |  | M3-M2 | 55.403 (18)^***^ | -.006 | .012 |
| Multigroup measurement invariance | | | | | | | | | | |
| Configural (M1) | 216.942 | 210 | .999 | .998 | .007  [.000, .017] |  |  |  |  |  |
| Metric (M2) | 241.577 | 224 | .998 | .995 | .010  [.000, .019] |  | M2-M1 | 24.498 (14)^*^ | -.001 | .003 |
| Scalar (M3) | 258.679 | 238 | .997 | .995 | .011  [.000, .019] |  | M3-M2 | 13.962 (14) | -.001 | .001 |

*Note*. M = model; χ^2^ = chi-square; df = degree of freedom; CFI = Comparative Fit Index; TLI = Tucker-Lewis Index; RMSEA = Root Mean Square Error

of Approximation; CI = confidence interval; Δ = change in the parameter.

^*^ *p* < .05; ^**^ *p* < .01; ^***^ *p* < .001

**S6. Latent Growth Curve Models: Model selection and results in the whole sample**

To examine mean-level changes in identification with the European group occurring in the medium- and short-term, a series of Latent Growth Curve models (LGCM) were applied. This strategy allows to estimate the mean levels (i.e., intercept) and rates of change (i.e., slope) of European identification, as well as the variability in these parameters. This analytic strategy implies fitting multiple models of increasing complexity (i.e., intercept-only, linear slope, non-linear slope) and comparing them against each other. Specifically, an intercept-only model (M1), which assumes full stability of the construct examined, was tested first and compared against a model that accounts for linear change (i.e., factor loadings are fixed, starting from 0 and increasing of one unit every time point; M2). In turn, this was compared against a non-linear model (M3) implying that identification changes with varying degrees over time (i.e., M*plus* was let free to estimate all factor loadings but two).

The quality of each model is evaluated based on a combination of fit indices. The Comparative Fit Index (CFI) and the Tucker–Lewis Index (TLI) with values higher than .90 and .95 indicate an acceptable and very good fit, respectively. The Root Mean Square Error of Approximation (RMSEA) with values below .08 and .05 are indicative of an acceptable and very good fit, respectively (Byrne, 2012), and the RMSEA’s 90% confidence interval’s upper bound lower than .10 indicates an acceptable fit of the model (Chen et al., 2008). When compared against each other, one model would be deemed significantly different from the other if at least two of the following three conditions were satisfied: a significant Δχ_SB_^2^ (Satorra & Bentler, 2001), and ΔCFI ≥ -.010 supplemented by ΔRMSEA ≥ .015 (Chen, 2007). Fit indices and results of model comparisons are reported in Table S6. As can be inferred, a free-change model was the best fitting solution to represent change in the medium-term, whereas linear models were found to be more suitable to describe short-term developmental patterns of identification with the European group.

**Table S6**

Latent Growth Curve Models: Model comparison

| Models | Model fit | | | | |  |  | Model comparisons | | |
| --- | --- | --- | --- | --- | --- | --- | --- | --- | --- | --- |
|  | χ^2^ | df | CFI | TLI | RMSEA [90% CI] |  | Models | Δχ_SB_^2^ | ΔCFI | ΔRMSEA |
| Monthly assessment (*N*=1,462) | | | | | | | | | | |
| Intercept-only (M1) | 111.483 | 26 | .932 | .945 | .047 [.039, .057] |  |  |  |  |  |
| Linear (M2) | 51.497 | 23 | .977 | .979 | .029 [.018, .040] |  | M1-M2 | 45.432(3)^***^ | -.045 | .018 |
| Free-change (M3)^1^ | **30.001** | **18** | **.991** | **.989** | **.021 [.005, .034]** |  | **M2-M3** | **19.148(5)^**^** | **-.014** | **.008** |
| Week 1 (*N*=509) | | | | | | | | | | |
| Intercept-only (M1) | 99.970 | 26 | .906 | .924 | .075 [.060, .091] |  |  |  |  |  |
| Linear (M2) | **66.432** | **23** | **.945** | **.950** | **.061 [.044, .078]** |  | **M1-M2** | **62.556(3)^***^** | **-.039** | **.014** |
| Free-change (M3) | NO CONVERGENCE | | | | |  | M2-M3 |  |  |  |
| Week 2 (*N*=525) | | | | | | | | | | |
| Intercept-only (M1) | 60.681 | 26 | .943 | .954 | .050 [.034, .067] |  |  |  |  |  |
| Linear (M2) | **37.928** | **23** | **.975** | **.977** | **.035 [.012, .055]** |  | **M1-M2** | **18.483(3)^***^** | **-.032** | **.015** |
| Free-change (M3)^1^ | 27.704 | 18 | .984 | .981 | .032 [.000, .054] |  | M2-M3 | 8.941(5) | -.009 | .003 |
| Week 3 (*N*=268) | | | | | | | | | | |
| Intercept-only (M1) | 63.764 | 26 | .844 | .874 | .074 [.051, .097] |  |  |  |  |  |
| Linear (M2) | **32.944** | **23** | **.959** | **.962** | **.040 [.000, .069]** |  | **M1-M2** | **20.597(3)^***^** | **-.115** | **.034** |
| Free-change (M3)^1^ | 45.491 | 18 | .886 | .867 | .075 [.048, .103] |  | M2-M3 | 5.346(5) | .073 | -.035 |

*Note*. M = model; χ^2^ = chi-square; df = degree of freedom; CFI = Comparative Fit Index; TLI = Tucker-Lewis Index; RMSEA = Root Mean Square Error of Approximation; CI = confidence interval; Δ = change in the parameter. ^1^The model with time 1 and 7 fixed was chosen.

^*^ *p* < .05; ^**^ *p* < .01; ^***^ *p* < .001

**S7. Latent Class Growth Analysis: Model selection procedure and results**

Latent Class Growth Analysis (LCGA) allows to identify within the population subgroups of individuals characterized by homogeneous developmental parameters. Models with an increasing number of classes were tested and compared against each other to identify the best fitting and most parsimonious solution. A combination of fit indices, parsimony, and theoretical meaningfulness is used to select the final solution.

Regarding fit indices, adding one class should result in improvements in model fit as evident from a decrease in the Sample Size Adjusted Bayesian Information Criteria (SSA-BIC; Sclove, 1987), a significant value of the adjusted Lo-Mendell-Rubin Likelihood Ratio Test (LMR-LRT; Lo et al., 2001), and an Entropy value equal or higher than .75 (Reinecke, 2006). Regarding parsimony, each subgroup identified by the LCGA should include at least 5% of the whole sample. Last, concerning theoretical meaningfulness, adolescents are expected to display either low, average, or high levels of identification with the European group and growth parameters (i.e., intercept and/or slope) should be significantly different across the three groups for meaningful interpretation of findings.

Results of the model selection procedure are detailed in Table S7. Although the four-class solution displayed a significant decrease in SSA-BIC, coupled with a significant LMR-LRT and a good Entropy score, the three-class solution was still deemed more appropriate for two reasons. First, one of the classes in the four-group solution included only 4% of the total sample. Second, in this model, the high identification class was split into two separate classes with both high intercepts (one slightly higher than the other) and equally increasing slopes, violating parsimony and making it hard to discern groups. Unstandardized growth parameter estimates for each class are reported in Table S8.

**Table S7**

Latent Class Growth Analysis: Model comparison

| Solution | SSA-BIC | Entropy | Adj. LMR-LRT | Trajectory group prevalence (%) | | | |
| --- | --- | --- | --- | --- | --- | --- | --- |
|  |  |  |  | 1 | 2 | 3 | 4 |
| 1-class solution | 14207.281 | - | - | 100 |  |  |  |
| 2-class solution | 12913.340 | .634 | 1249.138^***^ | 59 | 41 |  |  |
| 3-class solution | **12400.783** | **.709** | **501.932^*^** | **63** | **27** | **10** |  |
| 4-class solution | 12226.347 | .708 | 178.599^**^ | 53 | 35 | 8 | 4 |

*Note.* SSA-BIC = Sample Size Adjusted Bayesian Information Criteria; LMR-LRT = Lo-Mendel-Rubin

Likelihood Ratio Test.

^*^ *p* < .05; ^**^ *p* < .01; ^***^ *p* < .001

**Table S8**

Unstandardized growth parameters of the LCGA three-class solution

|  | Intercept | Slope |
| --- | --- | --- |
|  | *M* (SE) | *M* (SE) |
| Low identification group (10%) | 2.438^***^ (0.107) | -0.031^*^ (0.012) |
| Moderate identification group (63%) | 3.161^***^ (0.044) | 0.000 (0.005) |
| High identification group (27%) | 3.788^***^ (0.052) | 0.032^*^ (0.012) |

*Note.* *M* = Mean, SE = Standard Error, *σ^2^* = variance. Intercept and slope parameters of the three groups were all significantly different from each other based on Wald tests.

^*^ *p* < .05; ^***^ *p* < .001

**S9. Sensitivity Analyses**

Given that the total sample included both ethnic majority and minority participants, additional sensitivity checks were conducted by testing again the main models with the inclusion of adolescents’ background (0 = ethnic majority, 1 = ethnic minority) as covariate. These sensitivity analyses were not pre-registered and thus have an exploratory aim. Several analytical checks were conducted.

First, participants’ ethnic background was included as a covariate of medium- and short-term growth parameters (i.e., intercept and slope) in the Latent Growth Curve Models for the total sample, as well as in the multigroup version of the LGC models. Model fit indices and results are reported in Table S9. Regarding medium-term development, adolescents’ ethnic background was not significantly associated with the initial levels nor with the rates of change of European identification in the total sample. However, results of multigroup analyses revealed that being an ethnic majority was associated with slightly higher initial levels of identification with the European group in the younger compared to the older cohort of adolescents (Wald(df) = 8.115(1), *p* = .004). Regarding short-term development, adolescents’ ethnic background was not significantly linked to initial levels and rates of change in European identification in both the total sample and in multigroup analyses. It should be noted that including participants’ background as covariate in the LGC model of week 3 led to a not positive definite covariance matrix. Thus, model fit indices were not computed, and results should be interpreted with caution.

Second, adolescents’ ethnic background was included as covariate in the conditional LGC models (total sample and multigroup analyses) examining the interplay between medium-term developmental trajectories and short-term fluctuations. The main results were fully replicated. Moreover, ethnic background was not significantly associated with daily fluctuations in the total sample (*r* = -.033, *p* = .405) and across the two age groups (Younger cohort: *r* = -.041, *p* = .433; Older cohort: *r* = -.007, *p* = .912).

Last, the multinomial logistic regression model was tested again accounting for participants’ ethnic background among the auxiliary variables of class membership. Results are reported in Table S10. As can be inferred, adolescents’ ethnic background was not significantly associated with membership to one of the three developmental trajectories classes. However, when accounting for it, adolescents’ age became marginally significant (*p* = .051) in influencing the chances of being in the high compared to the low identification group.

**Table S9**

Latent Growth Curve Models with covariate: Model fit and standardized correlations

| Models | Model fit | | | | | Ethnic background ↔ | | | | | |
| --- | --- | --- | --- | --- | --- | --- | --- | --- | --- | --- | --- |
|  | χ^2^ | df | CFI | TLI | RMSEA [90% CI] | Int. | Slope | Int.  Y | Slope Y | Int.  O | Slope  O |
| Medium-term development | | | | | | | | | | | |
| Total sample | 36.650 | 23 | .990 | .988 | .020 [.005, .031] | .016 | .073 |  |  |  |  |
| Multigroup | 83.587 | 46 | .974 | .968 | .033 [.021, .043] |  |  | **.134^**^** | -.099 | **-.085** | .143 |
| short-term development | | | | | | | | | | | |
| Week 1 – Total sample | 72.815 | 28 | .952 | .952 | .032 [.023, .041] | -.039 | .062 |  |  |  |  |
| Week 1 – Multigroup | 97.267 | 56 | .957 | .957 | .031 [.020, .041] |  |  | .045 | .074 | -.087 | .078 |
| Week 2 – Total sample | 43.463 | 28 | .978 | .978 | .019 [.006, .029] | .046 | -.118 |  |  |  |  |
| Week 2 – Multigroup | 84.793 | 56 | .962 | .962 | .026 [.014, .037] |  |  | .025 | -.012 | .056 | -.191 |
| Week 3 – Total sample | NOT COMPUTED | | | | | -.053 | -.301 |  |  |  |  |

*Note*. M = model; χ^2^ = chi-square; df = degree of freedom; CFI = Comparative Fit Index; TLI = Tucker-Lewis Index; RMSEA = Root Mean Square Error of Approximation; CI = confidence interval; Int. = intercept parameter; Y = younger age cohort (1^st^ year students at T1); O = Older age cohort (3^rd^ year students at T1).

Bolded values indicate a significant difference (based on Wald test comparisons) in the correlation between participants’ ethnic background and growth parameter(s) for younger and older youth.

^**^ *p* < .01

**Table S10**

Multinomial logistic regression with covariates

|  | Average identification group vs. Low identification group | | High identification group vs. Low identification group | |
| --- | --- | --- | --- | --- |
| Predictors | *B* (*SE*) | OR  [95% C.I.] | *B* (*SE*) | OR  [95% C.I.] |
| Daily fluctuations | -0.928 (0.535) | 0.395  [0.139, 1.128] | -1.791^**^ (0.537) | 0.167  [0.058, 0.478] |
| Age group | -0.335 (0.323) | 0.715  [0.380, 1.348] | -0.633^†^ (0.325) | 0.531  [0.281, 1.003] |
| Ethnic background | -0.046  (0.441) | 0.955  [0.402, 2.264] | 0.175  (0.404) | 1.192  [0.540, 2.631] |

*Note.* Age group: 0 = younger, 1 = older; Ethnic background; 0 = majority, 1 = minority.

*B* = Unstandardized regression parameter; SE = Standard Error; OR = Odds Ratio; CI = Confidence Interval.

Grey-shaded cells contain results that became marginally significant when including covariates.

^†^*p* = .051; ^**^ *p* < .01
